# Zinc, type 2 diabetes, diabetic kidney disease: focus on hypoxia

**DOI:** 10.3389/fendo.2026.1825409

**Published:** 2026-07-21

**Authors:** Svetlana A. Lebedeva, Ekaterina M. Mikhailova, Maria Yu Materenchuk, Denis V. Kurkin, Mikhail Samsonov, Vadim V. Tarasov, Kerim Mutig

**Affiliations:** 1 Sechenov First Moscow State Medical University, Moscow, Russia; 2 Skolkovo Institute of Science and Technology, Moscow, Russia; 3 Center for Molecular and Cellular Biology, Moscow, Russia; 4 Russian University of Medicine, Moscow, Russia; 5 Medical Department, R-Pharm JSC, Moscow, Russia; 6 Scientific Center of Genetics and Life Sciences, Sirius University of Science and Technology, Sirius, Russia

**Keywords:** diabetes, diabetic kidney disease, HIF-1α, hypoxia, hypoxia-inducible factors, insulin, pancreatic β-cells, zinc

## Abstract

The incidence of type 2 diabetes mellitus is approaching pandemic levels, posing a major global healthcare problem. Diabetic kidney disease (DKD) is the main cause of the chronic kidney disease. Hyperglycemia-dependent renal hypoxia drives the onset and progression of DKD. The adaptive cellular response to hypoxia depends on the zinc signalling, which promotes expression of the hypoxia-inducible factor-1α (HIF-1α). Zinc is further relevant for anti-inflammatory, antioxidant, anti-apoptotic, and anti-fibrotic signalling pathways with nephroprotective effects in DKD. In this review, we focus on the role of zinc homeostasis in the development of diabetes and DKD, with particular emphasis on hypoxia as the central trigger of DKD. We also discuss the role of the zinc signaling in the cellular and systemic carbohydrate metabolism in the context of diabetes and DKD.

## Graphical Abstract

**Figure:**
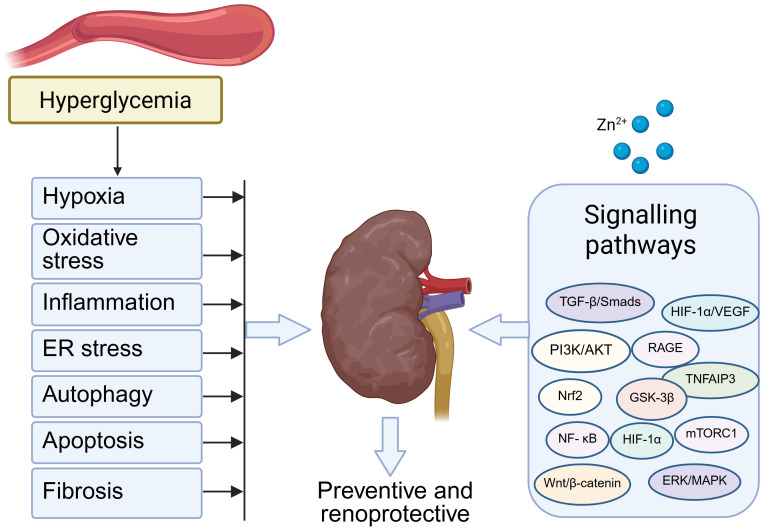

## Introduction

By 2045, projections suggest that nearly 640 million people worldwide will be affected by diabetes, reaching nearly pandemic levels (1). Type 2 diabetes mellitus (T2DM) is a multifactorial chronic metabolic disease, manifested by severe hyperglycemia, resulting from insulin resistance and/or impaired insulin secretion by pancreatic β-cells (2). Insulin resistance is defined as impaired insulin signalling and reduced biological responsiveness to insulin stimulation, predominantly in skeletal muscle, which accounts for approximately 80% of postprandial glucose disposal (3). In addition to skeletal muscle, the liver and adipose tissue are also major insulin-sensitive tissues.

Chronic hyperglycemia causes alterations not only in carbohydrate metabolism, but also in lipid and protein metabolism, as well as hypoxia, oxidative stress, and mitochondrial dysfunction. The accumulation of advanced glycation end-products (AGEs), lipid metabolites, peroxides, inflammatory mediators, and iron leads to functional impairment of target organs, including the kidneys, heart, and retina. As a result, T2DM can lead to severe complications such as diabetic kidney disease (DKD) (4–6), neuropathy, retinopathy, and diabetic foot ulcers. Approximately 30–40% of individuals with T2DM develop DKD (7). Renal hypoxia represents a fundamental pathogenetic mechanism underlying the onset and progression of DKD (8–11). Maintenance of zinc homeostasis is critically important for the organism’s ability to tolerate hypoxic conditions (12). Preserving optimal zinc levels may contribute to slowing down or even halting the progression of DKD.

The complex interplay between hyperglycemia, T2DM, the development and progression of DKD, and zinc metabolism has attracted increasing attention in recent years. Compared with healthy individuals, patients with T2DM exhibit hyperzincuria (13) and hypozincemia (14–17).

Zinc deficiency can impair the pancreatic insulin synthesis and secretion and lower glucose uptake in peripheral tissues, thereby contributing to insulin resistance (18). Nevertheless, the association between circulating zinc levels and the diabetes onset remains controversial, as evidence suggests that an increased risk of T2DM in individuals with isolated impaired glucose tolerance may be associated with elevated serum zinc levels (19). Taken together, these data indicate that both zinc deficiency or zinc excess, for example due to overdose of zinc supplements, may increase risk of diabetes, consistent with the U-shaped relationship between dietary zinc intake and diabetes risk. The inflection point of this relationship occurs at approximately 9.1 mg of zinc per day, whereas the lowest risk occurs with zinc intake ranging from 8.9 to 12.2 mg per day (20). Thus, epidemiological data suggest that the transition from a protective to a proapoptotic effect may occurat dietary zinc intake ≥12.2 mg/day (20), while at the cellular level, zinc toxicity begins at intracellular concentrations exceeding 100 µM, with apoptosis observed at concentrations of ~150 µM (21).

Systematic reviews and meta-analyses have demonstrated that zinc supplementation benefits individuals with diabetes or those at high risk of developing diabetes (prediabetes) by reducing blood glucose concentrations (22–24), glycated hemoglobin (HbA1c) (25–28), non-enzymatic glycosylation, and high-sensitivity C-reactive protein (hs-CRP) (26) (29),, while improving insulin responsiveness (24), and lipid profiles (22, 25, 27, 30, 31). However, the use of zinc supplements in T2DM patients requires more precise and effective preventive and therapeutic strategies regarding the optimal zinc form, dosage, and duration of administration (25), which are beyond the scope of the present review.

## The biological role and homeostasis of zinc

Zinc is the second most abundant trace element in the human body after iron, with the highest concentrations found in skeletal muscle (~60%) and bone (~30%). It serves as a cofactor for approximately 300 enzymes and a structural component of nearly 3000 proteins, accounting for around 10% of the human proteome (32). This trace element performs structural and catalytic functions, and also acts as a neurotransmitter involved in rapid cellular signalling (33, 34).

By inhibiting the activation of nuclear factor κB (NF-κB), zinc upregulates the expression of A20 and peroxisome proliferator-activated receptor-alpha (PPAR-α), both zinc-finger proteins with established anti-inflammatory functions (35). Zinc deficiency is associated with elevated levels of inflammatory biomarkers such as interleukin-6 (IL-6), tumor necrosis factor (TNF), and hs-CRP (36, 37). Beyond its anti-inflammatory effects, zinc exhibits antioxidant properties (38), enhancing glutathione and catalase activity, stabilizing protein sulfhydryl groups, and participating in redox reactions with highly reactive metals. Zinc also plays a critical role in tissue development, renewal, and repair, including the regulation of cellular proliferation and apoptosis (35). Moreover, zinc release in response to various stimuli initiates kinase–phosphatase cascades and modulates transcriptional regulation of genes (39).

Zinc plays a crucial role in mitochondrial generation of reactive oxygen species (ROS) under hypoxic exposure, causing phosphorylation and subsequent activation of nicotinamide adenine dinucleotide phosphate (NADPH) oxidase (40). By modulating the activity of hypoxia-inducible factor-1α (HIF-1α) through multiple mechanisms, zinc contributes to the organism’s adaptation to hypoxic conditions (12).

In its free form, zinc exists as Zn^2+^ ions, which are localized in the endoplasmic reticulum (ER), Golgi apparatus (GA), mitochondria, and specialized vesicles known as zincosomes. Because Zn^2+^ ions are hydrophilic, their transmembrane transport requires specialized transporters from two families: SLC30A (ZnT; Zn^2+^ transporters), which export zinc from the cytoplasm into the extracellular space or pump it into zincosomes and cellular organelles, and SLC39A (ZIP; Zrt/Irt-like protein), which increase intracellular zinc levels by importing it into the cytoplasm from extracellular and intracellular compartments (41–44). The expression levels of zinc transporters depend on tissue type, zinc concentration and physiological or pathological conditions.

Most intracellular zinc is protein-bound. By modulating protein activity, zinc acts as an intracellular signalling molecule at multiple levels of signal transduction, thereby influencing numerous metabolic pathways (45).

Approximately 20% of intracellular zinc is bound to metallothioneins (MTs), low-molecular-weight, cysteine-rich (~30%) proteins that act as metallochaperones and regulate zinc availability according to physiological needs (46, 47). In mammals, including humans, four MT isoforms (MT1, MT2, MT3 and MT4) are present, each capable of chelating up to seven zinc ions (48). MTs buffer zinc under physiological conditions and control its intracellular storage and release (49). Beyond their primary role in element homeostasis, MTs protect cells from oxidative stress induced by various factors, including toxic metals, and participate in cell survival, angiogenesis, vascular remodeling, immunomodulation and inhibition of apoptosis (50). The transcription of MT1 and MT2 genes is regulated by metal-responsive transcription factor-1 (MTF-1), a zinc-finger transcription factor that modulates the expression of metal-sensitive genes (51). In addition to regulating MT expression, MTF-1 induces the expression of the ZnT-1 gene, a membrane transporter responsible for exporting excess zinc from the cell, thereby preventing cytotoxicity (52). Among metals, only zinc is known to activate MTF-1. Interestingly, MTF-1 is also responsive to oxidative stress, reflecting its sensitivity to cellular redox changes (53).

Overall, cellular zinc homeostasis and bioavailability are tightly controlled by zinc transporters and MTs. The coordinated action of these mechanisms is essential for maintaining intracellular zinc homeostasis. Zinc transporters generate the “zinc wave”, providing a dynamic signalling mechanism, while MT-mediated buffering constitutes a relatively static protective system that maintains a stable zinc reserve and prevents zinc toxicity during prolonged elevations in zinc levels. The “zinc wave” promotes tubular cell proliferation, suppresses apoptosis of damaged cells, reduces inflammation and fibrosis, and stimulates angiogenesis by modulating signalling pathways. MTs provide a stable zinc reserve that supports repeated signalling waves, scavenge reactive oxygen species, thereby contributing to antioxidant defence, prevent zinc toxicity during sustained zinc elevation, and help preserve cellular homeostasis. However, zinc regulation under pathological conditions induced by hyperglycemia, including DKD, remains poorly understood and insufficiently characterized.

## Zinc transporters and β-cell function

The link between diabetes and zinc was first recognized nearly a century ago by D.A. Scott, who demonstrated that zinc is essential for insulin crystal formation (54), and that the pancreas of individuals with diabetes contains approximately half of the zinc content of healthy pancreata (55). Indeed, pancreatic β-cells contain markedly higher zinc concentrations than other cell types (56). Acting as an enzymatic cofactor, zinc is required at all stages of insulin synthesis, storage, and secretion (57, 58), and it stabilizes insulin in its hexameric form. In the context of glucose regulation, maintaining an adequate level of free zinc within β-cells is critical for insulin synthesis, secretion, stabilization, and storage. However, excessive intracellular zinc can induce apoptosis, highlighting the need for precise zinc homeostasis control.

### SLC30A/ZnT transporters

β-cells express a broad range of zinc transporters, with the most important belonging to the SLC30A (ZnT) family: ZnT3, ZnT5, ZnT6, ZnT7, and ZnT8. These transporters differ in intracellular localization and function. ZnT3 is localized in the membranes of insulin secretory granules and pumps zinc ions into them (59, 60). ZnT5, in addition to secretory granule membranes, is primarily found in the ER and GA (61), suggesting its role not only in the formation and stabilization of insulin–zinc complexes but also in intracellular zinc trafficking, particularly during the early stages of insulin granule formation and maturation (61, 62). ZnT7 and ZnT6 are predominantly localized in the GA and ER, although the precise distribution of ZnT6 remains incompletely characterized; it likely resides in multiple organelles. Both transporters contribute to maintaining zinc homeostasis and organelle function, processes essential for proper insulin synthesis and packaging.ZnT8 is embedded in the membrane of dense insulin granules where it mediates zinc accumulation, storage, and preparation of insulin for secretion (63). Unlike other transporters, ZnT8 exhibits a unique expression profile largely restricted to pancreatic islets, highlighting its central role in insulin processing, accumulation, and secretion.

**Figure 1:**
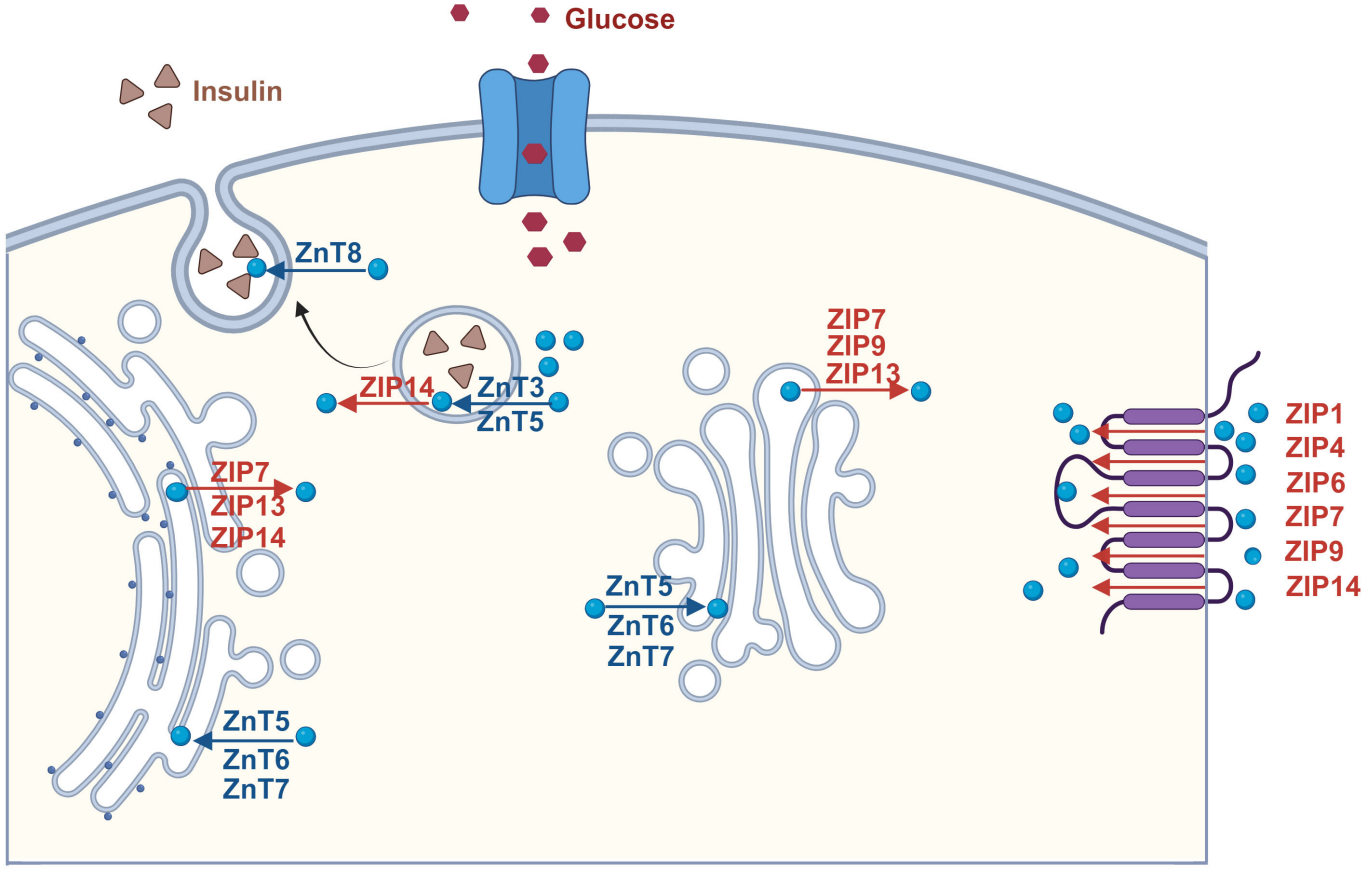
Localization of zinc transporters in pancreatic β-cells. ZnT3 is located in the membrane of insulin secretory granules and pumps zinc ions into them. ZnT5, ZnT6, and ZnT7 are typically localized in the membranes of the Golgi apparatus (GA) and endoplasmic reticulum (ER). ZnT8 is present in the membrane of dense-core insulin granules responsible for insulin accumulation, storage, and preparation for secretion. The role of ZnT transporters is to reduce cytosolic zinc by pumping it into the GA, ER, and insulin secretory granules. In contrast, ZIP transporters function to increase cytosolic zinc concentrations by importing it from the extracellular space into the cytosol and releasing it from intracellular organelles.

There is an inverse relationship between ZnT8 and ZnT3 expression: increased ZnT3 levels are associated with decreased ZnT8 expression, resulting in reduced insulin synthesis and secretion, and vice versa (59). Elevated ZnT3 expression in β-cells, observed under high glucose concentrations and zinc deficiency, indicates its involvement in adaptive responses to metabolic stress. In particular, ZnT3-knockout mice exhibit impaired glucose handling and disturbedcarbohydrate metabolism under metabolic stress (64). In pancreatic β-cells, increased ZnT3 expression reduces insulin stores and diminishes glucose-stimulated insulin secretion, as demonstrated in glucose-sensitive INS-1E β-cell lines (59). Interestingly, despite impaired insulin metabolism, increased cell viability and enhanced resistance to stress across varying glucose concentrations have also been reported. This negative effects of ZnT3 hyperexpression are mediated through suppression of ZnT8 expression, which plays a central role in insulin processing and secretion (**Figure 1**).

miRNA-mediated knockdown of ZnT3 and ZnT8 in INS-1E cells resulted in a significant reduction in insulin secretion and increased apoptosis (64). These findings underscore the essential role of ZnT3 in β-cell survival.

ZnT8 autoantibodies are detected in 60–80% of individuals with type 1 diabetes (T1DM) (65), reflecting an immune response directed against this zinc transporter and the autoimmune destruction of pancreatic β-cells. The polymorphic variant rs13266634 of the SLC30A8 gene encoding ZnT8 has been consistently associated with an increased risk of T2DM (66–70). However, a rare loss-of-function (LOF) allele in SLC30A8 (p.Arg138*) confers approximately a 65% reduction in T2DM risk in humans, likely by enhancing glucose sensitivity and reducing the activity of ATP-sensitive potassium channels (KATP) (71). Reduced KATP channel activity leads to membrane depolarization and subsequent activation of L-type calcium channels, promoting calcium influx and triggering glucose-stimulated insulin secretion. One proposed mechanism suggests that zinc, acting as an intracellular signalling molecule in β-cells, activates KATP channels at micromolar concentrations, thereby suppressing calcium channel opening and limiting insulin release. Thus, zinc-dependent modulation of KATP channels may provide a negative feedback loop during glucose-stimulated insulin secretion (72). Zinc may also compete with calcium in L-type channels through modulation of their activity and effects on ion fluxes via partial channel blockade or conformational alteration, reducing the calcium permeability. Such modulation does not represent classical ion competition for a single binding site but rather reflects modulation of the channel’s ion selectivity and gating properties.

Notably, studies have demonstrated that moderate physical exercise increases serum and pancreatic zinc levels, as well as pancreatic ZnT8 expression, which are reduced in streptozotocin-induced diabetic rats (73).

### SLC39A/ZIP transporters

Unlike SLC30A (ZnT) family, the role of the SLC39A (ZIP) in β-cell function is less well characterized (72). ZIP transporters mediate zinc influx into the cytosol from the extracellular compartment and intracellular organelles. The increased cytosolic zinc availability promotes zinc accumulation in insulin granules, thereby stimulating insulin secretion.

Mouse β-cells express ZIP1, ZIP4–9, ZIP13–14 (74, 75), indicating a link between zinc levels and normal β-cell function. ZIP4 up-regulation *in vitro* increases zinc accumulation in the cytosol and granules of beta cells, thereby stimulating insulin secretion. However, *in vivo* studies in mice have shown that ZIP4 is not essential for normal β-cell physiology and insulin secretion (76).

In three mouse models of diabetes, SLC39A5 was the only gene consistently downregulated in pancreatic β-cells (77). Whole-body SLC39A5-knockout mice show increased serum zinc accumulation and reduced blood glucose despite impaired glucose-stimulated insulin secretion (77). These findings support the idea that excess zinc lowers blood glucose primarily via insulin-mimetic effect rather than by enhancing insulin secretion (77). This process involves stimulation of glucose uptake and oxidation, increased lipogenesis from carbohydrates, and inhibition of lipolysis. In addition, zinc regulates glucose metabolism by directly modulating glucose transporters and key metabolic enzymes, including pyruvate dehydrogenase. By contrast, mice with SLC39A5 knocked out specifically in β-cells show impaired zinc uptake and defective glucose-stimulated insulin secretion. These defects are accompanied by dysregulation of the Sirt1–Pgc-1α signalling axis, reduced GLUT2 expression, and compromised ATP production (77). To date, no direct experimental evidence has demonstrated that zinc deficiency is a causal factor in the dysregulation of the Sirt1–Pgc−1α axis and associated mechanisms in human β−cells. Nonetheless, a biologically plausible link, together with substantial indirect evidence, suggests that zinc deficiency may contribute to the perturbation of these pathways and, consequently, to impaired glucose uptake and metabolism in β−cells.

Suppression of ZIP6 and ZIP7 expression reduces zinc influx into pancreatic cells, highlighting the importance of these transporters in maintaining cellular zinc levels and insulin release (78). ZIP14 is abundantly expressed in the ER of β-cells. Notably, SLC39A14 regulation in pancreatic islets is reduced in patients with T2DM (75). Phenotypically, mice with whole-body SLC39A14 knockout exhibit enlarged pancreatic islets with hyperinsulinaemia and increased body fat — conditions commonly associated with T2DM and obesity (79, 80). In hepatocytes, ZIP14 expression and zinc transport are increased during glucose uptake (80).

Thus, zinc participates in insulin synthesis, storage, and secretion, while zinc-transporting proteins contribute to intracellular signalling processes that regulate glycaemic control in peripheral tissues by modulating cytosolic zinc levels. Insulin production in β-cells clearly depends on zinc metabolism, which is tightly regulated by transporters and MT. However, the mechanisms by which modulation of transporter or MT activity confers protection against diabetes remain largely unknown. On the one hand, intracellular zinc protects cells from stress-induced apoptosis; on the other, increased zinc concentrations contribute to cellular dysfunction. These processes are challenging to study in rodent models, as they do not fully recapitulate the human phenotype or the protective effects observed in humans (71). This can be explained by several key differences, including gene haploinsufficiency versus complete knockout, phenotypic heterogeneity among mouse strains, and species-specific differences. For example, SLC30A8 knockout mice exhibit a marked reduction in β−cell zinc content but only a mild metabolic phenotype, whereas SLC30A8 haploinsufficiency is associated with protection against T2DM.

### ZnR/GPR39-mediated signalling in insulin secretion

While intracellular zinc regulation relies primarily on the MTs and SLC30A and SLC39A transporter families, the biological effects of extracellular zinc are mediated through the orphan G protein-coupled receptor GPR39. This receptor is widely expressed across endocrine and metabolic organs, including the intestine, stomach (81), spleen, lungs, liver, kidneys, adipose tissue, heart (82), pancreatic β-cells, and the epithelium of the exocrine ducts of the pancreas (81, 83, 84). GPR39 plays a role in regulating cell proliferation, differentiation, survival, and apoptosis, as well as in modulating inflammation and protecting against oxidative damage (85). Consequently, it is involved in the proper functioning of the nervous (82, 86–90), cardiovascular (91), immune (92), digestive (93), and reproductive systems (94, 95), as well as the liver and pancreas (96). Mechanistically, GPR39 activates Gαq, Gα, and Gα12/13 signalling pathways, and modulates some key inflammation signals, reducing tissue damage, promoting cell proliferation, and enhancing tissue repair. These properties makes it a promising therapeutic target for inflammatory diseases (96).

The affinity of GPR39 for zinc is regulated by Ca^2+^ (97), and the receptor itself serves as a link between fluctuations in extracellular Zn^2+^ concentrations and intracellular Ca^2+^ signalling (98). Increase in intracellular Ca^2+^ activates the extracellular signal-regulated kinase/mitogen-activated protein kinase (ERK/MAPK) and phosphoinositol-3-kinase/protein kinase B (PI3K/AKT signalling pathways (93), which in turn stimulate Na/H^+^ exchange isoform 1 (NHE1), K^+^/Cl^−^ cotransporters (KCC), and potentially the Na^+^K^+^2Cl^−^ cotransporter 1 (NKCC1) – ion transport systems essential in tissues expressing GPR39 (98).

Activation of NHE1 extrudes protons from the cell while importing sodium, leading to an increase in intracellular sodium concentration. Elevated sodium levels reduce the efficiency of the Na^+^/Ca^2+^ exchanger (NCX), resulting in intracellular Ca^2+^ accumulation. This rise in cytosolic calcium serves as a critical signal for pancreatic β-cells to secrete insulin. In addition, NHE1 activation alkalinizes the cytosol and alters the membrane potential, promoting the opening of voltage-dependent calcium channels (VDCC) and further calcium influx from the extracellular space. Thus, activating NHE1 activation in β-cells is essential for maintaining intracellular pH homeostasis, supporting proper insulin secretion, and ensuring overall β-cell function.

Data on KCC and NKCC1 expression in pancreatic cells remain limited. Available evidence indicates that NKCC1 is expressed in pancreatic islet β-cells in both human and mice, where it is implicated in ionic homeostasis and insulin secretion, whereas evidence supporting the expression of KCC isoforms in islets is considerably more limited and is derived mainly from broader KCC expression surveys rather than detailed islet−specific mapping. KCCs are thought to be primarily expressed in α-cells (99), while NKCC1 is present in β−cells of human and mouse islets and has been implicated in ionic regulation and insulin secretion (100).

**Figure 2:**
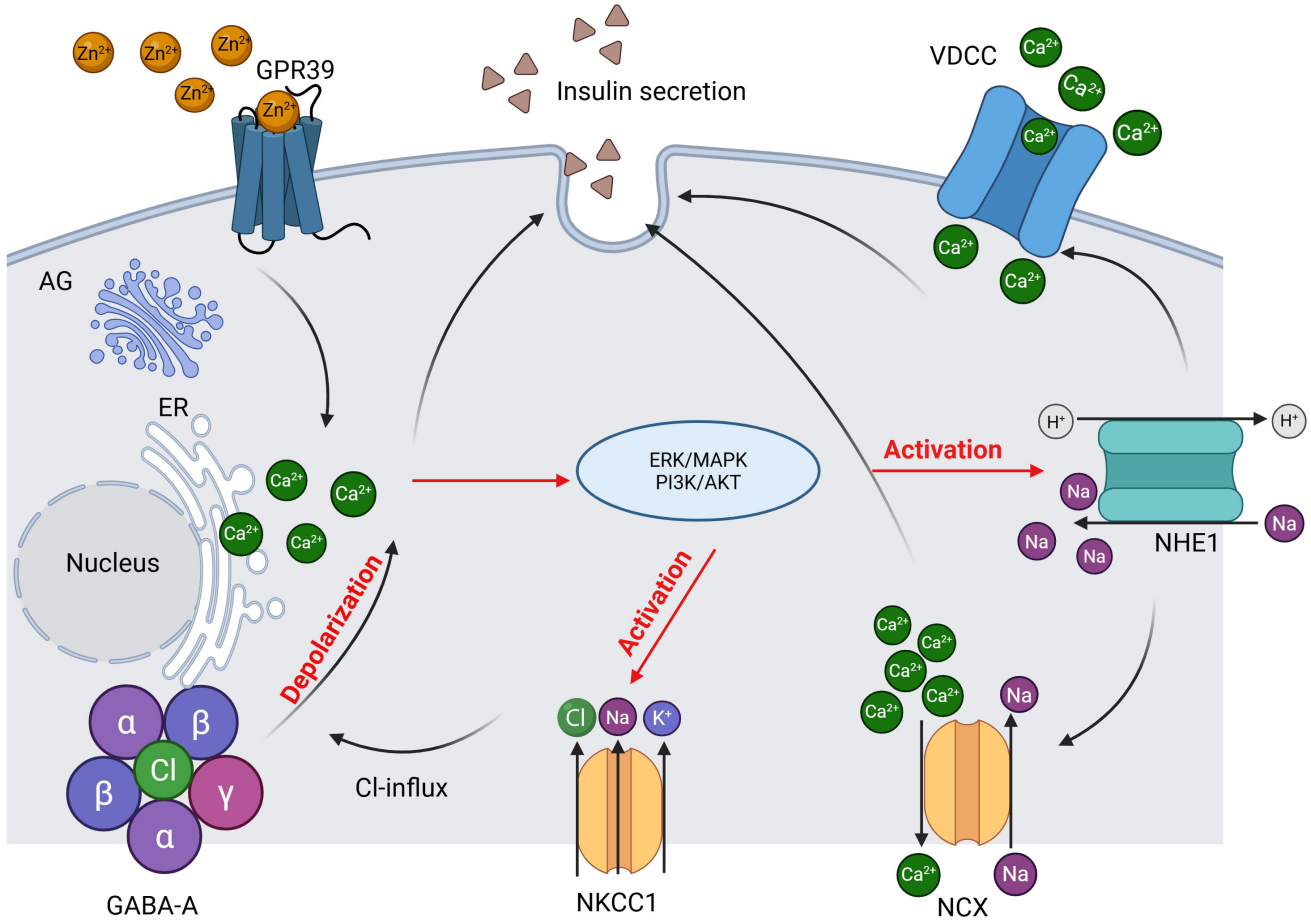
GPR39-dependent pathways associated with insulin secretion. Interaction of extracellular zinc with the GPR39 receptor leads to Ca^2+^ release from the ER and activation of the ERK/MAPK and PI3K/AKT signaling pathways. This results in activation of NHE1 and NKCC1 in pancreatic β-cells. Upon activation, NHE1 increases the extrusion of H^+^ from the cell in exchange for Na^+^ influx, leading to an increase in intracellular Na^+^ levels. This reduces the Na^+^ gradient normally used by the NCX exchanger to extrude Ca^2+^ from the cell. Reduced NCX efficiency leads to intracellular Ca^2+^ accumulation and enhanced insulin secretion. Activation of NHE1 also causes intracellular alkalinization and changes in membrane potential, promoting the opening of voltage-dependent calcium channels (VDCC) and additional Ca^2+^ influx from the extracellular space, which further increases insulin secretion. Activation of NKCC1 leads to intracellular accumulation of Na^+^, K^+^, and Cl^−^ ions. When intracellular chloride concentration is high, the chloride reversal potential may exceed the resting membrane potential, and Cl^−^ flux through GABA-A receptors may reverse direction. In this case, receptor activation leads to depolarization and an excitatory effect, which promotes activation of insulin secretion.

KCC activity in α-cells extrudes sodium and chloride ions, lowering the intracellular Cl^−^ concentration below the electrochemical equilibrium. This gradient activates gamma-aminobutyric acid А (GABA-A) receptor, allowing chloride influx, which hyperpolarizes the membrane, suppresses calcium signalling, and inhibits glucagon secretion.When intracellular chloride levels are elevated, the chloride reversal potential can exceed the resting membrane potential. Under these conditions, chloride flux through GABA-A receptors may reverse, resulting in depolarization rather than inhibition. Consequently, activation of GABA-A receptors in β-cells with high intracellular chloride can excite the cells, enhancing insulin secretion (**Figure 2**).

Overall, the interplay between GPR39 and ion transporters remains incompletely understood.

In the context of the pancreas endocrine function, zinc ions act as paracrine regulators of the islets of Langerhans by binding to GPR39. Insulin levels are significantly reduced in GPR39 knockout mice compared with controls (84, 101), whereas increased GPR39 expression in pancreatic β-cells protects against hyperglycemia (102). These findings demonstrate that GPR39 is essential for normal glucose tolerance in mice and plays a key role in the regulation of pancreatic function and glucose metabolism.

Beyond the pancreas, zinc action through GPR39 enhances ERK1/2 phosphorylation in the liver. In ZnR/GPR39 knockout mice, hepatic insulin receptor expression and AKT activation were increased. These animals exhibited elevated fasting glucose, enhanced hepatic lipid accumulation, increased levels of reactive oxygen species (ROS), and pronounced tissue fibrosis, reflected by significantly increased collagen expression compared with wild-type mice (103). These findings indicate that ZnR/GPR39 modulates insulin receptor signalling, highlighting its potential as a therapeutic target for the prevention and treatment of diabetes and related metabolic disorders (84).

Further investigation is required to elucidate the physiological functions of GPR39 and to enable effective targeted therapies for zinc-deficiency–associated conditions, including DKD. Despite abundant GPR39 expression in the kidneys, only a single study has examined its functional role in the organ. GPR39 localizes on the basolateral membrane of collecting duct epithelial cells, which also express the water transport protein aquaporin-2 (AQP2). Activation of GPR39 counteracts vasopressin by lowering the amount of AQP2 on the apical membrane, thereby decreasing water reabsorption in the collecting duct. In parallel, GPR39 activation increased K^+^ excretion through a selective reduction of phosphorylated Na^+^-Cl^−^ cotransporter in the distal convoluted tubule. Together, these findings indicate that GPR39 functions as a structural antagonist of vasopressin in regulating renal water transport, reducing vasopressin-induced water permeability and limiting the kidney’s ability to concentrate urine (104).

## Zinc and glucose metabolism

Glucose metabolism in humans comprises a complex network of interconnected biochemical processes aimed at obtaining, storing, and utilizing energy. Blood glucose homeostasis is maintained by a neurohormonal system that integrates hormonal and neural mechanisms. Hormones interact to form an insulin–glucagon index, which determines the predominance of glycogen synthesis or breakdown depending on the organism’s energetic state. Insulin, secreted by pancreatic β-cells, promotes glucose uptake into muscle and adipose tissue, enhances glycolysis and glycogen synthesis, and suppresses gluconeogenesis and glycogenolysis, thereby lowering blood glucose levels. In contrast, glucagon, secreted by α-cells in response to low glucose, activates hepatic glycogenolysis and gluconeogenesis, raising blood glucose. Adrenaline acts similarly to glucagon, primarily in muscle, stimulating glycogenolysis, whereas cortisol promotes gluconeogenesis and glycogen synthesis in the liver while reducing glucose utilization by peripheral tissues. At the CNS level, the hypothalamus coordinates neurohormonal signals via both neural pathways and hormonal regulation. Molecularly, glucose metabolism is regulated through transcriptional and post-translational mechanisms that modulate the activity of gluconeogenic enzymes, as well as proteins such as signal transducer and activator of transcription 3 (STAT3), which promote glucose utilization through gene activation. Thus, glucose metabolism is a dynamically regulated process that ensures a continuous energy supply to cells while maintaining blood glucose within a narrow physiological range.

The role of zinc in cellular signalling pathways that influence glucose homeostasis is currently under active investigation. In addition to its effects on insulin synthesis, secretion, and storage, described above, several potential mechanisms of zinc’s anti-hyperglycemic action have been proposed, some of which mimic insulin-like signalling.

Acting as a secondary messenger, zinc inhibits the activity of phosphatase and tensin homolog deleted on chromosome 10 (PTEN), leading to increased PIP3 levels and activation of the insulin signalling pathway mediated by PI3K/AKT (105–108).

Intracellular zinc also inhibits protein tyrosine phosphatase 1B (PTP1B), which normally dephosphorylates and deactivates the insulin receptor. This inhibition enhances the activation of the insulin receptor tyrosine kinase domain, amplifies insulin signal transduction, and increases insulin sensitivity. The activated insulin receptor tyrosine kinase phosphorylates tyrosine residues on insulin receptor substrate 1 (IRS1) and insulin receptor substrate 2 (IRS2), recruiting SH2-domain-containing proteins, including PI3K, thereby initiating an intracellular signalling cascade that mediates the physiological effects of insulin (109).

In addition to the PI3K/AKT pathway, which mediates the rapid (short-term) effects of insulin, zinc is involved in a second signalling mechanism that mediates slower (long-term) insulin effects. This mechanism is mediated via the ERK/MAPK signalling cascades and is associated with altered expression of nuclear genes regulating cellular growth, proliferation, and differentiation. The MAPK/ERK pathway has been shown to act through the transcription factor Ets-1, which controls the expression of the insulin-like receptor gene (110).

Of particular interest is the study of zinc’s effects on insulin signalling in normal and insulin-resistant skeletal muscle cells, as skeletal muscle consumes nearly 80% of ingested glucose (111) and represents the primary site of peripheral insulin resistance (112). In insulin-resistant skeletal muscle, defects in glucose metabolism include reduced insulin receptor tyrosine phosphorylation, decreased AKT phosphorylation, and impaired GLUT4 translocation. Notably, these insulin signalling targets are also activated by zinc treatment in skeletal muscle (111).

**Figure 3:**
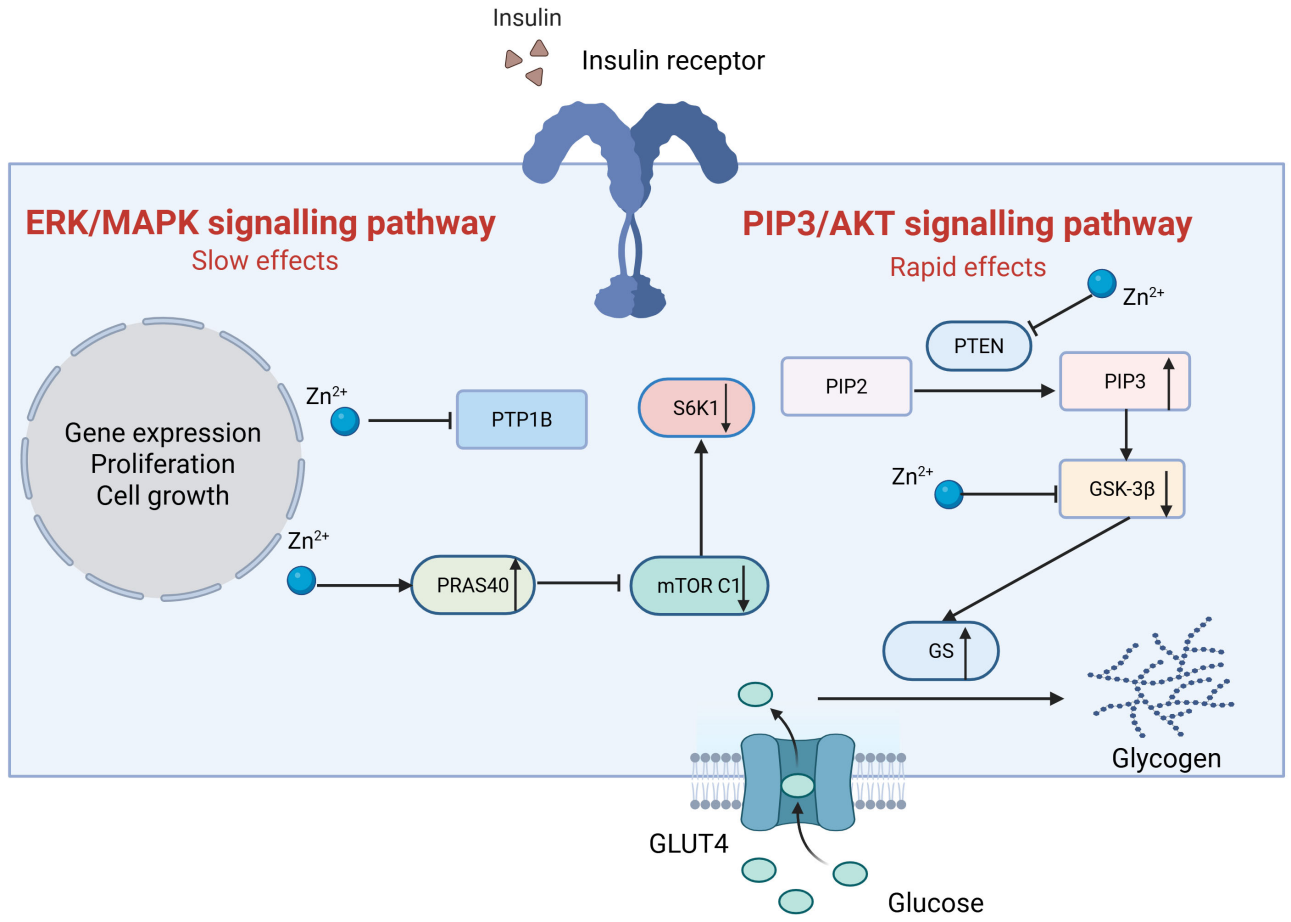
The role of zinc in cellular insulin signalling. Zinc modulates insulin signaling by inhibiting PTEN and PTP1B, thereby sustaining insulin receptor phosphorylation and enhancing PI3K/AKT pathway activity. Activation of AKT may promote phosphorylation of PRAS40, relieving its inhibitory effect on mTORC1 and simultaneously inhibiting GSK-3β. These effects favor glycogen synthase activation and glycogen synthesis. In addition, attenuation of the mTORC1/S6K1 axis may reduce the inhibitory serine phosphorylation of IRS-1, thereby helping to preserve insulin sensitivity.

In human and murine skeletal muscle cell lines, zinc has been shown to increase ERK expression, thereby enhancing insulin signalling (109). Zinc also upregulates proline-rich AKT substrate of 40 kDa (PRAS40), which exerts inhibitory effects on the mammalian target of rapamycin complex 1 (mTORC1) (113), a major negative regulator of autophagy (114). Under normal insulin pathway activation, mTORC1 promotes protein synthesis, cell growth, and suppression of autophagy in skeletal muscle, contributing to the maintenance of muscle mass. However, excessive mTORC1 activation, which can be caused, for example, by high dietary intake of carbohydrates or fats, hyperinsulinemia, or inflammatory and oxidative stress, leads to activation of p70 ribosomal S6 kinase (S6K1) (115), S6K1 phosphorylates IRS-1 on serine residues, resulting in its inactivation, impaired interaction with the insulin receptor, diminished insulin signalling, and the development of insulin resistance (116). Notably, specific inhibition of S6K1 improves glucose tolerance and insulin signalling in obese mice by enhancing AKT phosphorylation in metabolic tissues, representing a potential therapeutic strategy for treating obesity-associated T2DM (117) (**Figure 3**).

Physiological concentrations of zinc have also been shown to inhibit glycogen synthase kinase-3β (GSK-3β), a serine/threonine protein kinase that plays a key role in the development of insulin resistance and T2DM (109, 118). Under normal conditions, insulin inactivates GSK-3β via phosphorylation, which is required for activation of glycogen synthase (GS), the enzyme responsible for converting glucose into glycogen in target cells such as skeletal muscle. In insulin-resistant states, GSK-3β inhibition is impaired, hindering glycogen synthesis and contributing to elevated blood glucose levels (119, 120).

GSK-3β can also phosphorylate IRS-1 on serine residues, leading to its inactivation and reduced cellular insulin sensitivity. Animal and human studies have shown that both the expression and activity of GSK-3β are elevated in skeletal muscle in T2DM and obesity (121), which is associated with impaired glycogen synthesis and decreased glucose uptake (120). In insulin-resistant states, GSK-3β activity is also increased in β-cells, resulting in reduced proliferation, enhanced apoptosis, and consequently, decreased β-cell mass, thereby exacerbating insulin deficiency in T2DM (120, 122). Inhibition of the non-phosphorylated form of GSK-3 improves insulin-stimulated glucose transport by enhancing insulin signalling and promoting translocation of glucose transporter type 4 (GLUT-4) vesicles to the plasma membrane (118). Zinc increases glucose uptake into cells (106, 123), enhances GLUT-4 expression, and regulates gluconeogenic enzymes such as glucose-6-phosphatase (G6Pase) and phosphoenolpyruvate carboxykinase (PEPCK), thereby improving overall glucose metabolism (123).

Furthermore, zinc ions have been shown to increase glucose uptake threefold in isolated mouse adipocytes, an effect comparable to that of saturated insulin concentrations (118).

Overall, these findings indicate that zinc exerts insulin-mimetic actions by enhancing glucose uptake in skeletal muscle cells (109, 111) and by activating key signalling molecules involved in the regulation of glucose homeostasis. Disruption of zinc homeostasis contributes to the pathogenesis of various chronic metabolic disorders, most notably diabetes and its associated complications.

## Zinc and diabetic kidney disease

### Pathophysiology of DKD

As a result of prolonged hyperglycemia, approximately 30-40% of patients develop DKD, a severe microvascular complication (7). DKD is characterized by hemodynamic alterations, metabolic disturbances, excessive accumulation of extracellular matrix (ECM), inflammation and fibrosis (124). Chronic hyperglycemia represents the primary driving force in DKD pathogenesis, triggering a cascade of renal abnormalities, including alterations in glomerular filtration rate (GFR), glomerulosclerosis, dysregulated tubuloglomerular feedback, tubule hypertrophy, renal hypoxia, podocyte injury, albuminuria, and lipotoxicity (125–127). Development of tubulointerstitial fibrosis is a key predictor of the progression from DKD to end-stage renal disease (ESRD) (128).

Even with adequate glycemic control, DKD may continue to progress. This may be attributable to “metabolic memory”, referring to the long-term consequences of prior hyperglycemic exposure on both macrovascular and microvascular complications of diabetes. Metabolic memory encompasses several interrelated mechanisms, including oxidative stress, inflammation, mitochondrial dysfunction, and epigenetic alterations such as DNA methylation, histone modifications, and non-coding RNAs (129, 130). Zinc may modulate these processes by affecting the antioxidant system, inflammatory responses, and genomic stability. However, the extent to which zinc influences epigenetic modifications remains unclear. It is plausible that zinc deficiency shifts epigenetic regulation toward a more stress−associated or pro−inflammatory profile. From this perspective, prevention of zinc deficiency may represent a potential prophylactic strategy. However, it should be noted that, at present, no fully effective strategies exist to halt the development or progression of DKD.

Renal hypoxia is considered a fundamental pathogenetic mechanism underlying both the onset and progression of DKD (8–11). Experimental studies in rats have demonstrated that renal tissue hypoxia develops very early in diabetes. A significant reduction in renal oxygen partial pressure is detected as early as two days after diabetes induction (131).

Despite receiving approximately 20% of the cardiac output, second only to the heart, the kidneys are intrinsically vulnerable to hypoxic injury (132). Since GFR and renal blood flow are tightly coupled, increases in renal blood flow do not proportionally enhance arterial oxygen delivery. Moreover, renal pO_2_ is maintained within a narrow yet stable range, spanning from approximately 5 mmHg in the medulla to about 50 mmHg in the cortex. In addition, renal arterio-venous oxygen shunting further aggravates oxygen availability in the medulla. As a result, this region receives only about 10% of the renal blood flow compared to the cortex, rendering it especially susceptible to hypoxia. Disruptions of the finely tuned balance between renal blood flow, GFR, oxygen consumption, and arteriovenous oxygen bypass lead to hypoxic kidney injury (133). The proximal part of the nephron tubules is characterized by high metabolic demand and is particularly vulnerable to hypoxic damage (134).

Hyperglycemia increases both glomerular filtration and the tubular reabsorption of glucose and sodium. These processes happen in the proximal tubule cells via the sodium-glucose linked transporters (SGLT1, SGLT2), which is highly ATP-dependent (10). Enhanced proximal sodium reabsorption reduces sodium delivery to the macula densa (MD) in the thick ascending limb. In response to decreased luminal sodium concentration, the MD activates tubuloglomerular feedback (TGF), leading to increased glomerular filtration and stimulation of renin and angiotensin II release (135). It leads to vasoconstriction of renal arterioles, which further impairs oxygen delivery within the renal tubular interstitium (136). In the DKD settings, the kidney, which is already highly susceptible to hypoxia, experiences a growing disparity between renal ATP demand and ATP generation. This metabolic imbalance exacerbates hypoxic injury, ultimately promoting disease progression (137).

Considering that most of the oxygen entering the kidneys is consumed by Na-K-ATPase, which mediates tubular reabsorption of nearly 99% of the filtered sodium (138), renal oxygen availability becomes critically limited. Mathematical modeling studies have shown that renal oxygen demand in diabetes may increase by up to 100% (139). However, diabetes-induced microvascular damage and impaired renal perfusion restrict oxygen delivery. Developing renal hypoxia alters the function of the cytochrome chain, resulting inreduced ATP production and increased ROS generation, thereby promoting mitochondrial dysfunction and oxidative stress. In the presence of hyperglycaemia, excessive ROS accelerate non-enzymatic glycation of proteins and lipids with the formation of AGEs. Binding of AGEs to receptors for advanced glycation end products (RAGE) activates intracellular signalling cascades that induce pro-inflammatory cytokine production, further enhance ROS generation and increase lipid peroxidation, thereby establishing a “vicious circle”. This processes cause damage to cellular membranes, proteins and DNA, increases endothelial dysfunction and inflammation, and ultimately contribute to the progression of diabetic vascular and tissue complications.

Chronic renal hypoxia in DKD damages the renal tubular interstitium, causing an imbalance between ECM synthesis and degradation, resulting in renal fibrosis (10).

Activation of the polyol pathway is connected to these pathological processes, which leads to the intracellular accumulation of osmotically active sorbitol and fructose. This induces osmotic stress, cellular swelling and functional impairment. At the same time, excessive consumption of NADH leads to an increased ratio of NADH/NAD^+^, a condition called pseudohypoxia, since the partial pressure of oxygen in tissues remains normal (140). In parallel, polyol pathway activation impairs the restoration of glutathione, a key intracellular antioxidant. This exacerbates oxidative stress, which both triggers and results from autophagic cell dysfunction (141). In addition, NADH deficiency reduces nitric oxide synthesis, impairing endothelial function and contributing to vascular damage.

Thus, sustained hyperglycaemia promotes hypoxia and amplifies oxidative stress by enhancing ROS generation (142), establishing a self-perpetuating cycle that leads to long-term kidney damage.

### Hypoxia responses

To adapt to hypoxic conditions, cells have developed complex mechanisms, the key among which is the activation of hypoxia-inducible factor (HIF). HIF is a pleiotropic, oxygen–sensitive transcription factor belonging to a broader group of transcription factors comprising the basic helix-loop-helix-PAS family and plays a central role in cellular adaptation to hypoxia.

HIF is a heterodimer composed of a labile α-subunit (HIF-1a, HIF-2a, or HIF-3α) and a common stables β subunit, which are 2 helix-loop-helix proteins. Upon activation, the heterodimer translocates to the nucleus, where in cooperation with co-activators, it induces the expression of numerous target genes. HIF directly activates hypoxia-responsive genes by binding to conserved hypoxia-response elements (HREs) within their regulatory regions.

HIF-1α is widely expressed across almost all body tissues, whereas HIF-2α’s exhibits a more restricted expression pattern, being predominantly found in endothelial cells of blood vessels, as well as in the kidney, liver, and brain. The abundance of α-subunit is regulated by cellular oxygen availability, while the β-subunit is constitutively expressed and oxygen-independent. Under normal oxygen levels, HIFs are degraded by Fe^2+^-dependent prolyl hydroxylases (PHD). Hydroxylated HIF−1α binds to the von Hippel–Lindau (pVHL) ubiquitin complex and is subsequently targeted for proteasomal degradation.

HIF-1α is important during the initial drop in oxygen levels, as it helps cells to adapt and survive. It also supports cellular energy production under hypoxic conditions. HIF-2α and HIF-3α play a larger role during prolonged hypoxia. Under low-oxygen conditions, HIF-2α stimulates erythropoietin production, which helps prevent cell death and protects cells from hypoxic damage.

HIF-1α and HIF-2α show distinct expression patterns in the kidneys. HIF-1α is weakly expressed in the outer cortex but strongly expressed in certain tubular and collecting duct epithelial cells.

HIF-2α is localized to the glomeruli, with dense nuclear staining in podocytes and microvascular endothelial cells (143, 144). Studies in mice with proximal tubule‐specific knockout of HIF‐1α (PT‐HIF‐1α^−/−^mice) showed worsened kidney damage in streptozotocin-induced diabetes, highlights the protective role of HIF-1α (145). Interestingly, HIF-1α is minimally expressed in healthy kidneys, but its expression is increased in DKD (146). HIF-1α levels are influenced not only by hypoxia but also by insulin regulation, insulin-like growth factors, AGEs, and blood sugar levels (10).

Reduced oxygen availability in diabetic patients requires the adaptive metabolic reprogramming from mitochondrial oxidative phosphorylation to glycolysis (147), a process in which HIF-1α plays a key role (146).

The *in vivo* and *in vitro* data support a molecular model in which glucose induces a HIF-1α-driven metabolic program via regulation of HIF-1α-target genes (146). HIFs can activate the transcription of over 100 genes, including vascular endothelial growth factor (VEGF), glucose transporter (GLUTs), erythropoietin (EPO), transforming growth factor-β1 (TGF-β1), and heme oxygenase-1 (HO-1). These genes confer protection against kidney damage under hypoxic conditions by promoting angiogenesis, modulating inflammation, enhancing glycolysis, and maintaining mitochondrial integrity (148). In particular, several studies have demonstrated that many renoprotective effects of SGLT2 inhibitors may be related to their ability to promote hypoxia signalling in the diabetic kidney (149, 150).

HIF-1α, the key regulator of adaptation to hypoxia, may protect the kidneys through several mechanisms, including promoting angiogenesis, increasing EPO production, reducing oxidative stress and inflammation, regulating programmed cell death, enhancing mitochondrial autophagy, and preventing fibrosis and vascular calcification (10).

Under prolonged hypoxia, the balance between HIF-1α and HIF-2α can be disrupted. Specifically, activation of HIF-1α coupled with suppression of HIF-2α can exacerbate inflammation and fibrosis in glomerular and renal tubular cells (149, 151).

**Figure 4:**
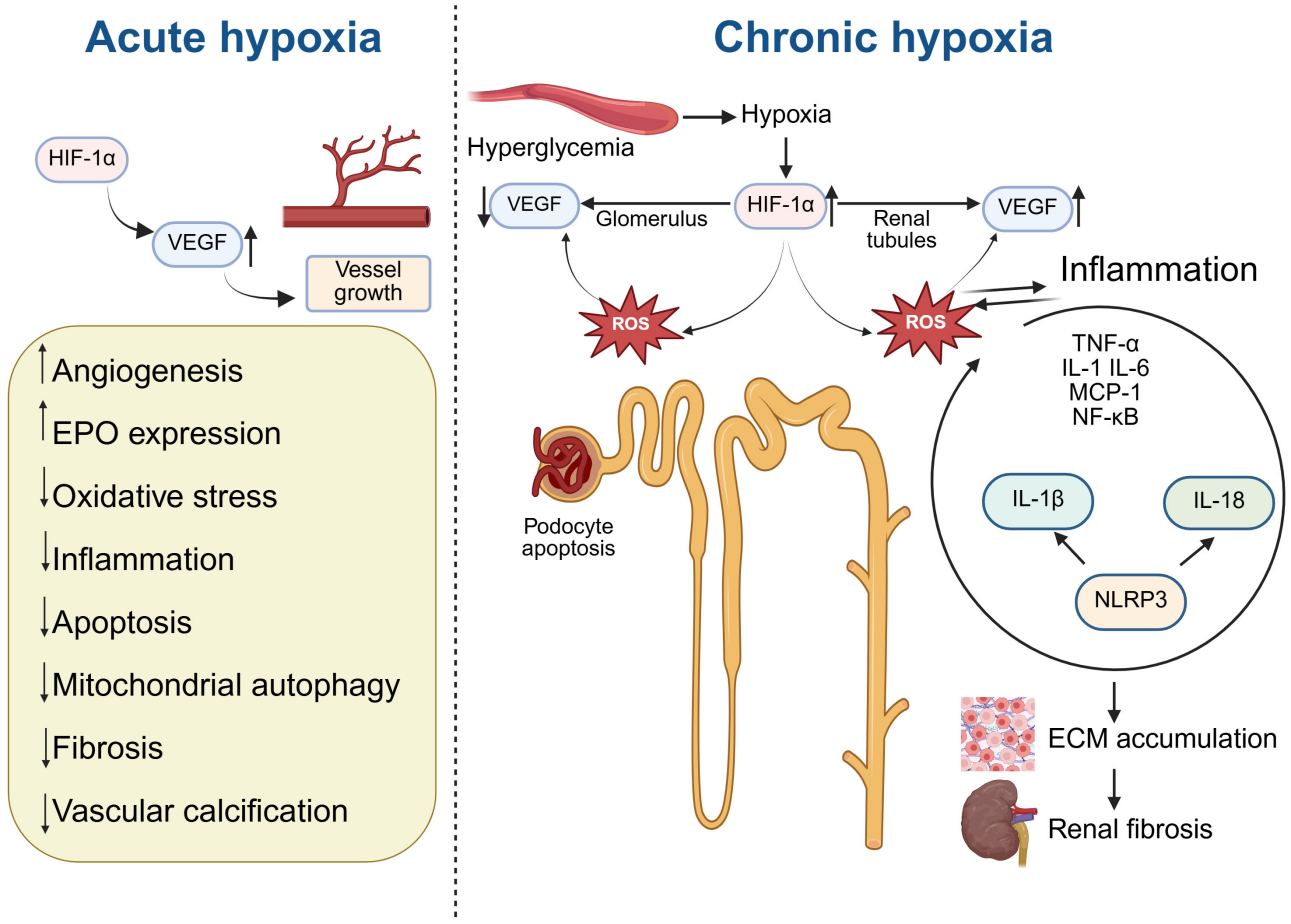
The role of HIF-1α in the kidney during acute and chronic hypoxia. During acute hypoxia, activation of HIF-1α initiates mechanisms aimed at protecting the kidney under oxygen deficiency. During chronic hypoxia, excessive activation (overexpression) of HIF-1α occurs along with suppression of HIF-2α expression. In the glomeruli, the hyperglycemic environment and hypoxia lead to increased ROS production and reduced VEGF expression and angiogenesis, resulting in podocyte apoptosis. In renal tubules, chronic hypoxia leads to VEGF overexpression and pathological angiogenesis, accumulation of ECM in the interstitial space, formation of fibrotic tissue, and progression of renal fibrosis. Overexpression of HIF-1α and enhanced ROS production increase the levels of pro-inflammatory cytokines, creating a vicious cycle of hypoxia and inflammation in the kidneys.

HIF-1α overexpression induces epithelial-mesenchymal transition (EMT) through pathways that upregulate lysyl oxidase 2 (LOXL2) and TGF-β1, leading to increased ECM production and renal interstitial fibrosis (RIF). Excessive HIF-1a activity also promotes the proliferation of fibroblasts and capillaries adjacent to kidney tubules by elevated VEGF production, resulting in ECM accumulation in the peritubular and pericapillary spaces. This process eventually forms fibrous tissue characterized by a dense capillary network, contributing to the progression of renal fibrosis (10). Overall, in DKD, HIF-1α overexpression exacerbates RIF, by driving EMT, promoting pathological angiogenesis, and increasing the expression of profibrotic factors (10) (**Figure 4**).

Thus, acute activation of HIF−1α is adaptive and reversible, initiating transcriptional programs to restore energy homeostasis and improve tissue oxygenation in response to transient hypoxia. In contrast, chronic overactivation is pathological, as it leads to persistent expression of genes involved in angiogenesis, cell proliferation, and metabolic reprogramming, and promotes chronic inflammation and tissue dysfunction.

HIF-1α activation in DKD may have dual effects. It enables kidney cells adapt to hypoxia, potentially protecting the tissue by regulating genes involved in blood vessel growth, metabolism, and antioxidant defence. However, chronic overactivation of HIF-1α in DKD can exacerbate inflammation and fibrosis, promoting further kidney damage. Targeted modulation of HIF-1α activity is currently considered a promising therapeutic strategy to minimize renal injury and enhance renoprotection.

### The role of zinc in the pathophysiology of DKD

As DKD progresses, plasma zinc levels decline (26, 152–154). Low zinc levels are negatively correlated with the expression of nod-like receptor nucleotide-binding domain and leucine-rich repeat pyrin-3 domain (NLRP3), IL-1β, and IL-18 (155) and are currently considered a risk factor for the development of chronic kidney disease (CKD) or the progression of kidney injury (7, 156–158). A retrospective cohort study using the TriNetX Analytics Network demonstrated that zinc deficiency serves as an independent predictor of DKD and affects kidney function through direct mechanisms rather than via microvascular dysfunction or poor metabolic control (159). This finding is particularly important, as it indicates that zinc deficiency represents an independent therapeutic target rather than merely a marker of overall diabetes severity.

A 2025 study using Mendelian randomization (MR), based on Mendel’s laws of inheritance, combined with proteomic analysis, identified 26 plasma proteins and four key signalling or metabolic pathways: digestion, events associated with the phagocytolytic activity of polymorphonuclear (PMN) cells, digestion and absorption, and DSCAM interactions, all of which mediate the association between decreased serum zinc levels and the development of DKD (160). Several factors may contribute to reduced plasma zinc levels, including decreased intestinal zinc absorption (41), drug interactions (161) and increased urinary zinc excretion (152).

The causes and mechanisms of zinc loss with urine during CKD remain unknown. Despite the well-established links between zinc deficiency and kidney disorders, data on the renal distribution of zinc transporters along the nephron and their cellular localization are still limited (158, 162). ZnT-1 has been reported to localize to the basolateral membrane of distal tubular epithelial cells (163) and, together with ZIP10, to participate in zinc reabsorption (164). Downregulation of ZnT-1 and ZIP10 in distal tubules may lead to reduced zinc efflux from tubular cells into the blood, contributing to hypozincemia, as well as impaired zinc reabsorption, potentially resulting in hyperzincuria, respectively. It remains difficult to determine whether decreased expression of ZnT-1 and ZIP10 is a primary cause or a secondary consequence of initial podocyte injury. It is likely, that zinc dysregulation represents both a cause and a consequence of podocyte damage: hyperglycemia induces initial podocyte injury and subsequently disrupts the expression of zinc transporters (including ZnT-1 and ZIP10) in the tubules, which in turn may further exacerbate injury and establish a self-amplifying pathological loop.

Expression of ZnT3, ZnT4, ZnT5, and ZnT6 genes has also been observed in pig kidneys, although the specific cell types were not identified (165).

ZnT7 is localized in the perinuclear region and GA of rat kidney tubular epithelial cells. Overexpression of ZnT7 inhibited high-glucose (HG)-induced EMT, whereas knockdown of ZnT7 promoted EMT. Furthermore, ZnT7 knockdown and the associated increase in HG-induced EMT were accompanied by activation of the MAPK/ERK and TGF-β/Smad pathways (166). It has also been shown that SLC30A7 exerts antioxidant effects in glucose-induced apoptosis via the NFE2L2/HMOX1 signalling pathway (167).

The kidneys are the second organ after the pancreas in terms of high ZnT8 expression (168). ZnT8 is predominantly expressed in renal tubular epithelial cells but only weakly in the glomerulus and podocytes (169). Studies indicate that ZnT8 protects against EMT and tubulointerstitial fibrosis by restraining the activation of the TGF-β1/Smads signalling pathway in DKD (169). Additionally, ZnT8 exerts protective effects against apoptosis of renal tubular epithelial cells through induction of TNF-α–induced protein 3 (TNFAIP3) and subsequent suppression of the NF-κB pathway (170).

Since plasma zinc and proteinuria are correlated (158) and albumin serves as a carrier for zinc in the blood, urinary albumin wasting may contribute to zinc deficiency frequently reported in CKD patients (162). The negative correlation found between urinary uromodulin excretion and the fractional excretion (FE) of zinc suggests that the urinary zinc losses may be linked to the low number of functional tubuli in CKD (158). Improved understanding of molecular pathways involved in renal zinc handling is mandatory for developing strategies to prevent zinc wasting in patients with CKD.

The role of zinc in the development and progression of CKD is not yet fully understood. Overall, it is thought to be related to the critical role of zinc in maintaining glomerular barrier function and supporting antioxidant defense systems, which protect against hyperglycemia-induced kidney damage (7, 171, 172). It is believed that most pathological changes leading to kidney injury, including abnormal renal hemodynamics, thickening of the glomerular basement membrane, mesangial expansion, extracellular matrix accumulation, and glomerulosclerosis, are largely driven by diabetes-induced ROS, providing a rationale for therapies targeting increased oxidative stress (173).

The key role of zinc in reducing oxidative stress, which is considered the common denominator of all mechanisms responsible for the progression of kidney diseases, has been described in numerous studies (7, 174). Oxidative stress in the kidneys is one of the main pathogenic mechanisms underlying the development of DKD. Zinc, due to its antioxidant properties, helps maintain redox balance and prevents cellular damage caused by hyperglycemia-induced oxidative stress. Zinc deficiency leads to increased oxidative stress and accumulation of free radicals.

Zinc-mediated activation of MTs protects the kidneys from oxidative stress by reducing ROS production (175). The observation that MT expression is increased in proximal tubule cells, which are most sensitive to oxygen deficiency, may also underscore the important role of MTs under hypoxic conditions (176).

Zinc induces MT expression in renal tubular cells *in vitro* (177), and *in vivo* studies have shown a significant positive correlation between MT levels and eGFR following zinc administration, suggesting a protective role of MT under conditions of impaired kidney function, potentially mediated by zinc (178).

The antioxidant mechanism of zinc may be associated with the induction of nuclear factor-erythroid 2-related factor 2 (Nrf2), a potent intracellular antioxidant that provides critical defense against ROS by activating superoxide dismutase (SOD), glutathione S-transferase, and other neutralizing agents. Nrf2 not only regulates cellular antioxidant activity but also participates in anti-apoptotic processes via the Wnt/β-catenin signalling pathway (179) and in EMT. Suppression of Nrf2 signalling in the context of elevated ROS has been observed in zinc-deficient mice (172). Additionally, patients with DKD exhibit reduced Nrf2 expression (152, 179).

Hypoxia developing in the kidneys during DKD is a major trigger of oxidative stress. Zinc plays a key role in adapting the organism to a hypoxic environment (12), by modulating HIF-1α activity through various mechanisms. In particular, molecular mechanisms investigations have demonstrated that zinc induce HIF-1α proteasomal degradation (180).

**Figure 5:**
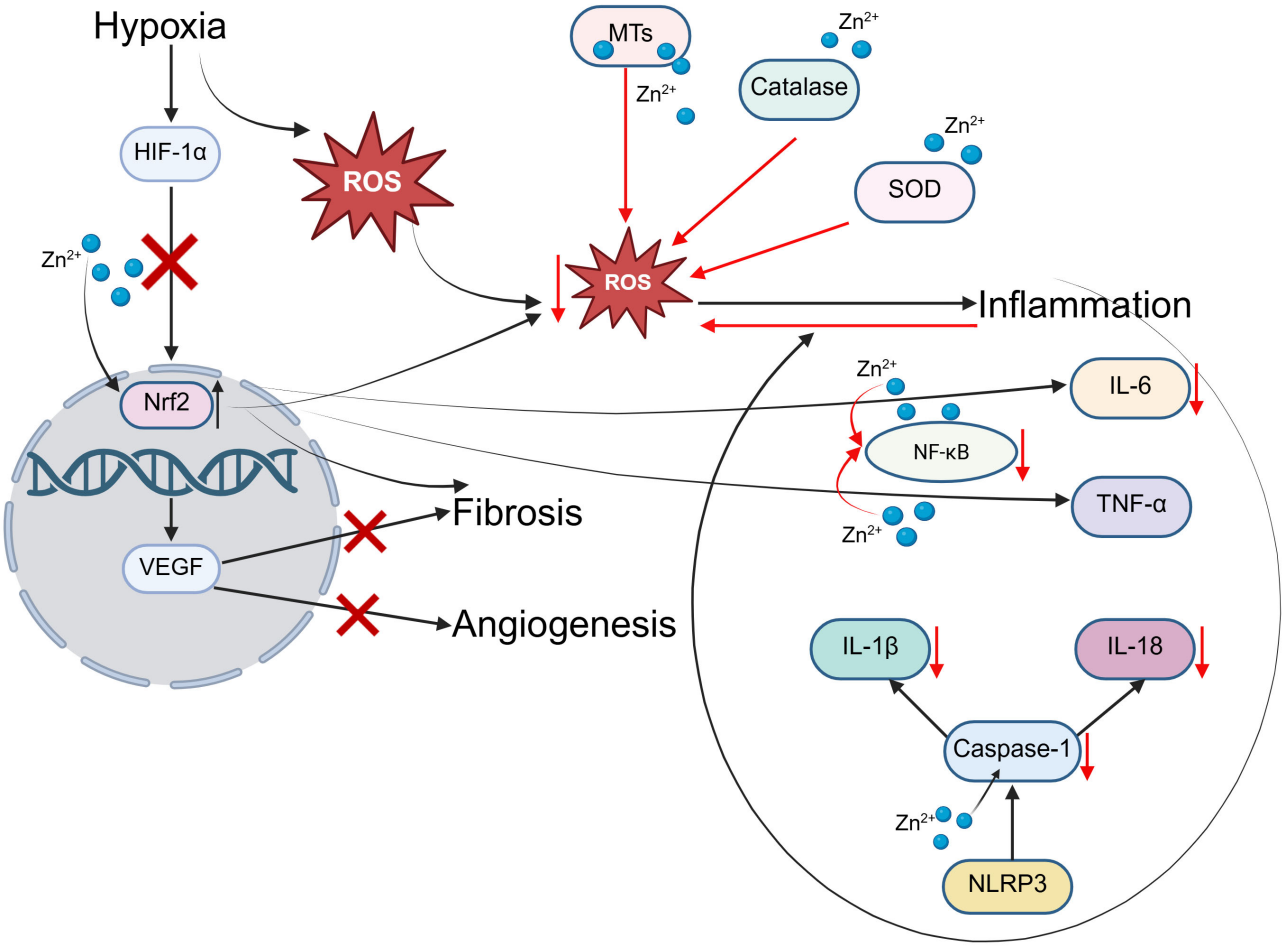
Zinc-dependent hypoxic pathways in DKD. Hypoxia activates HIF-1α, but zinc inhibits HIF-1α translocation to the cell nucleus for activation of target gene transcription. Zinc-mediated inhibition of HIF-1α nuclear translocation reduces activation of the HIF-1α/VEGF signaling pathway and decreases angiogenesis, fibrosis, and inflammation. Zinc activates Nrf2, enhancing antioxidant defense via SOD and catalase, thereby reducing ROS formation. Under oxidative stress, MTs release zinc to activate protective antioxidant pathways. Zinc inhibits the NF-κB pathway and suppresses production of IL-6 and TNF-α. Zinc suppresses NLRP3 inflammasome activation, reducing inflammation under oxidative stress conditions. Zinc directly binds to caspase-1, inhibiting secretion of IL-1β/IL-18.

Moreover, zinc inhibits the translocation of HIF-1α to the cell nucleus, preventing its binding to DNA and activation of target gene transcription. This may reduce activation of the HIF-1α/VEGF signalling pathway and slow fibrotic processes by decreasing angiogenesis and inflammation (**Figure 5**).

In animal models of DKD, zinc administration reduced the expression of fibrotic markers (181), tubular EMT, and tubulointerstitial fibrosis via activation of the PI3K/AKT/GSK-3β signalling pathway, accompanied by decreased regulation of HIF-1α expression (182). Modulation of the HIF-1α/VEGF signalling pathway represents a promising mechanism for controlling renal fibrosis and protecting kidney tissue from irreversible damage.

Zinc deficiency may disrupt the regulation of HIF−1α via several interrelated mechanisms, leading to a dysregulated state in which adaptive responses are impaired while pathological signalling is enhanced. The precise mechanisms by which zinc deficiency shifts the balance toward chronic pathological HIF-1α activity remain incompletely understood.

We hypothesize that zinc deficiency may impair pVHL-mediated proteasomal degradation of HIF-1α, leading to its persistent stabilization and nuclear accumulation. This results in sustained expression of HIF-1α target genes, including VEGF, GLUT1, and EPO, and promotes suppression of apoptosis, pathological angiogenesis, and fibrosis. In parallel, reduced activity of zinc−dependent antioxidant enzymes under zinc deficiency may lead to accumulation of ROS and chronic inflammation.

## Conclusion

Zinc is a fundamental trace element influencing both risk factors and the progression of DKD. Through its pleiotropic actions, zinc modulates multiple signalling pathways aimed at preventing DKD. It affects HIF-1α expression in cells exposed to hyperglycemic and hypoxic conditions via several mechanisms, including inhibition of HIF-1α translocation, and also exhibits anti-inflammatory, antioxidant, anti-apoptotic, and anti-fibrotic properties. Zinc deficiency is an independent predictor of adverse outcomes in T2DM and may serve as a potential biomarker for DKD risk stratification. Assessing zinc levels could improve risk stratification algorithms, potentially identifying patients at high risk for intensified monitoring and early intervention before the onset of clinical manifestations.
